# Quantitative Navigated Transcranial Magnetic Stimulation Motor Mapping and nTMS-Guided Corticospinal Tractography in Healthy Individuals and Patients with Motor-Eloquent Brain Tumors

**DOI:** 10.3390/brainsci16090986

**Published:** 2026-09-17

**Authors:** Evangelia Chatzikyriakou, Ioannis Vlachos, Konstantinos Pastiadis, Nikolaos Foroglou, Konstantinos Kouskouras, Georgios Feretos, Christos Frantzidis, Ioannis Patsalas, Vasileios Papaliagkas, Dimitris Kugiumtzis, Vasilios K. Kimiskidis

**Affiliations:** 1 First Department of Neurology, AHEPA University Hospital, Aristotle University of Thessaloniki, 54636 Thessaloniki, Greece; evangecg@auth.gr (E.C.); iovlachos@auth.gr (I.V.); 2 School of Music Studies, Aristotle University of Thessaloniki, 57001 Thessaloniki, Greece; pastiadi@mus.auth.gr; 3 Department of Neurosurgery, AHEPA University Hospital, Aristotle University of Thessaloniki, 54636 Thessaloniki, Greece; nforoglou@auth.gr (N.F.); g.feretos@gmail.com (G.F.); patsalas@auth.gr (I.P.); 4 Department of Radiology, Aristotle University of Thessaloniki, 54636 Thessaloniki, Greece; coskou@auth.gr; 5 School of Engineering and Physical Sciences, University of Lincoln, Brayford Pool, Lincoln LN6 7TS, UK; cfrantzidis@lincoln.ac.uk; 6 Department of Biomedical Sciences, International Hellenic University, 57001 Thessaloniki, Greece; vpapaliagkas@ihu.gr; 7 Department of Electrical and Computer Engineering, Aristotle University of Thessaloniki, 54124 Thessaloniki, Greece; dkugiu@auth.gr

**Keywords:** navigated transcranial magnetic stimulation, motor mapping, motor-eloquent brain tumor, motor-evoked potential, corticospinal tract, diffusion tensor imaging, tractography, neuroplasticity, presurgical mapping

## Abstract

**Highlights:**

**What are the main findings?**

- Patients showed impaired motor performance and suprathreshold corticospinal output, while rMT did not differ significantly from healthy controls.
- Patient–control differences extended to the contralateral hemisphere, and patients showed greater between-subject CoG dispersion.

**What are the implications of the main findings?**

- Threshold-based excitability measures alone may not capture the full functional impact of motor-eloquent brain tumors.
- Independent healthy controls may reveal motor system abnormalities not apparent from within-patient hemispheric comparisons.

**Abstract:**

Background/Objectives: Navigated transcranial magnetic stimulation (nTMS) enables presurgical motor mapping, while nTMS-seeded diffusion tensor imaging (DTI) delineates the corticospinal tract (CST). We compared cortical and tractography measures between healthy individuals and patients with motor-eloquent brain tumors and between patient hemispheres. Methods: Twenty healthy volunteers and 18 patients underwent first dorsal interosseous (FDI) mapping at 110% resting motor threshold (rMT). Motor map metrics, maximum FDI motor-evoked potential (MEP) amplitude, and nTMS-seeded CST measures were analyzed; hotspot and center-of-gravity (CoG) dispersion were assessed in common space. DTI metrics were available for 13 healthy volunteers and 13 patients. Results: Consistent with previous nTMS literature, patients showed impaired motor performance and suprathreshold corticospinal output, with lower grip strength, maximum FDI MEP amplitude, and map volume than controls. rMT did not differ significantly, indicating that threshold excitability may be unrevealing despite motor system dysfunction. Notably, the contralateral patient hemisphere also differed from controls in grip strength, maximum FDI MEP amplitude, reconstructed CST fiber length, and mean axial diffusivity, while these measures did not differ significantly between the ipsilateral and contralateral patient hemispheres. CoG dispersion was greater in patients, whereas hotspot dispersion was not. Conclusions: The study confirms tumor-associated impairment of motor performance and suprathreshold corticospinal output while identifying two potentially novel observations: abnormalities extending to the apparently unaffected hemisphere and increased heterogeneity of the amplitude-weighted motor map center. Notably, contralateral abnormalities may reflect bilateral network changes or patient-related factors, rather than isolated contralateral CST pathology. Independent healthy controls may therefore provide information not captured by within-patient hemispheric comparisons.

## 1. Introduction

Transcranial magnetic stimulation (TMS) enables non-invasive assessment of human cortical excitability and corticospinal output. When combined with MRI-based neuronavigation, navigated TMS (nTMS) provides spatially resolved functional motor maps that can be integrated into the presurgical evaluation of lesions involving or approaching motor-eloquent cortex [1,2]. Comparisons with intraoperative direct electrical stimulation (DES), together with systematic reviews and clinical cohort studies, support the utility of nTMS for localizing motor function, refining surgical strategy, and potentially improving the balance between extent of resection and preservation of motor function [3,4,5,6,7].

A central parameter in motor mapping is the resting motor threshold (rMT), which reflects the stimulation intensity required to evoke a predefined motor response in a relaxed target muscle. In conventional TMS practice, a positive MEP is commonly defined by a peak-to-peak amplitude of at least 50 μV, and threshold estimation seeks the intensity associated with an approximately 50% response probability [8]. Adaptive threshold-hunting procedures can estimate rMT efficiently and reproducibly [9,10]. Physiologically, rMT reflects the minimum stimulation required to elicit a predefined motor response, primarily indicating the excitability of cortical and corticospinal elements most readily activated. In contrast, suprathreshold stimulation involves recruitment and synchronization of a broader corticospinal neuron population, as well as downstream spinal mechanisms. Therefore, changes in motor system output may appear in suprathreshold MEP responses and motor map features without necessarily changing rMT. In patients with motor-eloquent tumors, excitability measures may be influenced by lesion location, histology, edema, medication, age, and the functional status of the motor system; therefore, a normal or abnormal rMT should not be interpreted in isolation.

nTMS can also provide functionally informed cortical seed regions for DTI tractography of the CST. This combined nTMS-DTI approach is used to visualize the spatial relationship among the motor cortex, descending motor pathways, and the lesion, thereby supporting cortical and subcortical risk assessment [11,12,13]. Nevertheless, deterministic DTI tractography is sensitive to acquisition quality, crossing fibers, edema, infiltration, and the choice of tracking thresholds. Quantitative tractography measures should therefore be interpreted as model-dependent imaging metrics rather than direct measures of axonal integrity.

Against this background, the present study had two primary aims: (1) to compare quantitative nTMS motor map and nTMS-DTI metrics between healthy volunteers and patients with tumors involving or adjacent to motor cortex; and (2) within the patient cohort, to compare the tumor-bearing and contralateral hemispheres and examine associations with clinical motor status. We hypothesized that patients would show altered motor output and spatial organization, whereas threshold-based mapping and tractography measures might remain technically feasible despite motor system abnormalities.

## 2. Materials and Methods

### 2.1. Population

This single-center, cross-sectional, observational study included 20 healthy volunteers and 18 patients diagnosed with CNS neoplastic lesions adjacent to or within the motor cortex, enabling comparison of motor mapping metrics across groups.

Patients were recruited from the hospital’s Department of Neurosurgery and referred to the Department of Clinical Neurophysiology for preoperative motor mapping as part of their clinical assessment. Healthy volunteers—comprising both right- and left-handed individuals to reduce hemispheric bias and to better match the patient cohort, given tumor lateralization across hemispheres—were recruited from hospital staff.

Inclusion criteria were: (1) age ≥ 18 years; (2) absence of contraindications to TMS as outlined by Rossi et al. [14]; and (3) adequate cognitive capacity to understand the study and to provide written informed consent.

Exclusion criteria for healthy volunteers were: (1) history of neurological or psychiatric disorders, including substance use disorder; (2) pregnancy; (3) use of centrally acting medications; and (4) metal implants (e.g., pacemakers, orthopedic plates). Participants reported sleeping 7–9 h before TMS, abstaining from alcohol for 24 h, and maintaining their usual caffeine intake. Handedness was assessed with the Edinburgh Handedness Inventory (EHI).

Exclusion criteria for patients were: (1) contraindications to TMS; (2) drug-resistant epilepsy; (3) inability to adhere to the protocol; and (4) use of benzodiazepines (other antiseizure medications at stable doses were permitted).

Before the experiment, all participants were screened for TMS contraindications using a detailed questionnaire [14]. Written informed consent was obtained from all subjects. The study was approved by the institutional ethics committee of Aristotle University of Thessaloniki (2.73/27 February 2019) and conducted in accordance with the Declaration of Helsinki.

### 2.2. Magnetic Resonance Imaging

All subjects underwent structural 3D T1-weighted brain MRI on a 1.5 T Philips Multiva scanner (Philips, Amsterdam, The Netherlands) (structural T1-weighted MPRAGE, 1 mm isotropic voxels, acquisition matrix 256 × 126).

Diffusion tensor imaging (DTI) was performed on a 1.5 T Philips Multiva scanner using 15 diffusion-weighted directions and one non-zero b-value (b = 1000 s/mm^2^), together with one b0 image. The acquired voxel dimensions/in-plane resolution were 2.21 × 2.28 mm, reconstructed to 1.25 × 1.25 mm, with a slice thickness of 4 mm and 55 slices. A parallel-imaging factor of 1.8 was used. No motion or eddy current correction was applied during preprocessing.

### 2.3. Navigated TMS, Electromyography, and Motor-Threshold Determination

Navigated TMS was performed using the eXimia NBS system (Nexstim Plc, Helsinki, Finland) with MRI-based neuronavigation and a figure-of-eight stimulation coil. Participants were seated comfortably and instructed to keep the tested hand relaxed and their eyes open. Surface electromyography (EMG) was recorded from the first dorsal interosseous (FDI) muscle using the integrated EMG system (3 kHz sampling rate; 10–500 Hz band-pass filter). Bipolar Ag/AgCl surface electrodes (0.6 cm^2^; 3M Red Dot) were placed in a standard belly–tendon montage, with the ground electrode at the ipsilateral wrist. Trials with background noise or preactivation > 20 μV peak-to-peak were excluded.

The anatomically defined primary motor cortex near the hand knob was explored with suprathreshold stimuli to identify the site producing the largest resting FDI motor-evoked potential (MEP), which was defined as the hotspot. At this site, coil position and orientation were adjusted tangentially to maximize the FDI MEP response.

Resting motor threshold (rMT) was estimated at the FDI hotspot using the maximum-likelihood adaptive threshold-hunting algorithm implemented in the Nexstim NBS software (version 6), consistent with adaptive threshold-hunting approaches [9,10]. A positive FDI response was defined as a peak-to-peak MEP amplitude ≥ 50 μV within a latency window of 17–30 ms. Threshold estimation ended when the predefined software convergence criterion (±2.5% of maximum stimulator output around the estimate) was reached or after a maximum of 30 stimuli.

### 2.4. nTMS Cortical Mapping

Following rMT determination, FDI motor mapping was performed manually at 110% rMT. Mapping began at the hotspot and proceeded outward over the surrounding motor cortex until the responsive region was completely encircled by two rows of non-responsive stimulation sites. An interstimulus interval of at least 5 s was maintained. Coil position and orientation were continuously monitored with neuronavigation and adjusted according to the local cortical anatomy.

In healthy participants, the dominant hemisphere was mapped. In patients, both hemispheres were mapped during the same session using the same procedure. MEP peak-to-peak amplitudes and stimulation coordinates were exported for quantitative analysis.

All nTMS examinations were performed by the same examiner, who was not blinded to the participants’ clinical status.

### 2.5. nTMS–DTI Integration

Deterministic, region-of-interest (ROI)-based corticospinal tract (CST) tractography was performed with Brainance DTI Software Suite v3.5.0 (Advantis Medical Imaging, Eindhoven, The Netherlands).

For CST reconstruction, two ROIs were used. The first ROI was derived from the motor map generated by TMS mapping, incorporating the stimulation points associated with motor responses, while the second ROI was manually delineated at the level of the pons based on anatomical landmarks. Only streamlines passing through both ROIs were retained for analysis. Tractography was performed using an FA threshold of 75% of the individual fractional anisotropy threshold (FAT75), following the approach proposed by Frey et al. [15]. The individual FAT was determined as the highest FA value at which a tract could still be reconstructed while satisfying the predefined minimum fiber length criterion. The final tractography was then performed at 75% of this individualized FAT with an angular threshold of 27°, a step size of 1 mm, and a streamline length range of 0–200 mm.

### 2.6. Muscle Cortical Representation

FDI muscle cortical representation (MCR) maps were constructed with TMSmap software v1.9.0.44b [16]. Map area (cm^2^), map volume (uV·cm^2^), hotspot coordinates, and center-of-gravity (CoG) coordinates were derived from the exported stimulation coordinates and MEP amplitudes. For quantitative topographical analysis, the initial online hotspot identified during the nTMS examination and used for rMT determination was not used as the final analytical hotspot. Instead, the hotspot was recalculated offline using TMSmap and defined as the cortical location at which the averaged MEPs exhibited the highest peak-to-peak amplitude [17]. The CoG was calculated as the amplitude-weighted spatial average of the motor-positive stimulation sites. Thus, both hotspot and CoG coordinates used for the subsequent spatial analyses were derived from the complete motor map rather than from the initial online hotspot determination.

**Figure 1:**
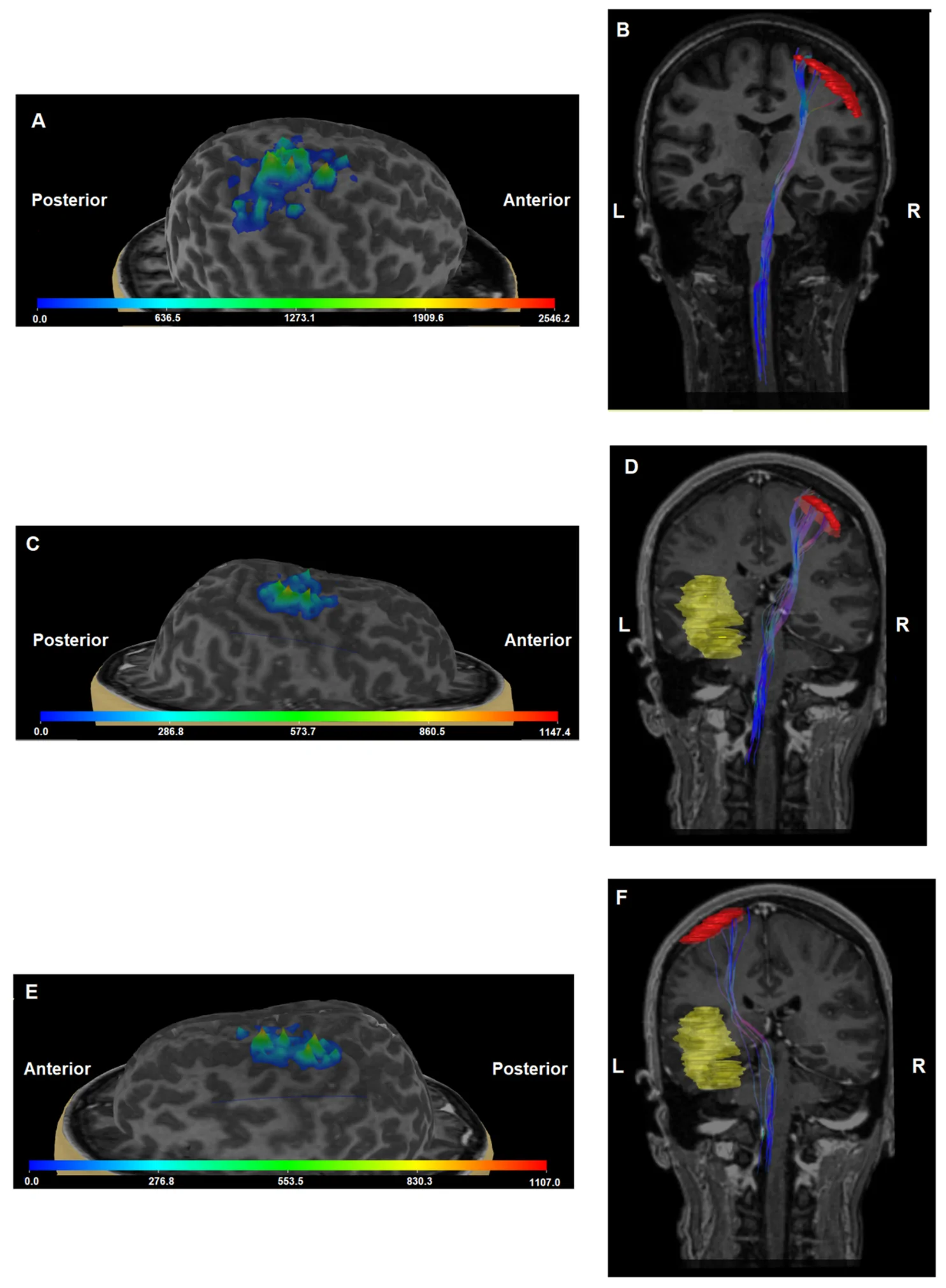
Representative 3D motor maps generated using TMSmap software (**A**,**C**,**E**) and 3D reconstructions of the somatotopic nTMS-DTI of the corticospinal tract (**B**,**D**,**F**): a healthy participant mapped in the dominant right hemisphere (**A**,**B**); an age- and sex-matched patient mapped in the unaffected right hemisphere (**C**,**D**); and the same patient mapped in the affected left hemisphere (**E**,**F**). In panels (**A**,**C**,**E**), the color scale represents MEP peak-to-peak amplitude (μV) and is individually scaled for each participant, ranging from 0 μV (blue) to the maximum MEP amplitude recorded for that participant (red). In panels (**B**,**D**,**F**), red indicates the nTMS motor map, whereas yellow represents the tumor. L: left; R: right.

Figure 1 illustrates representative 3D motor maps from a healthy participant and a patient.

### 2.7. Outcome Measures and Assessment

Outcome variables. Eleven quantitative outcomes were evaluated. Clinical and nTMS motor mapping outcomes comprised grip strength (kg), maximum FDI MEP amplitude, FDI map area, FDI map volume, and rMT. nTMS-DTI outcomes comprised number of fibers (NoF), mean fractional anisotropy (FA), mean axial diffusivity (AD), mean radial diffusivity (RD), mean fiber length, and FAT75. Spatial organization of the FDI representation was assessed separately using the hotspot and amplitude-weighted center of gravity (CoG).

Statistical comparisons. Three complementary analyses were performed. First, healthy controls and patients were compared using linear models adjusted for age and sex; adjusted marginal means are reported, and the condition effect is summarized by the adjusted standardized mean difference. Second, associations between the quantitative outcomes and clinical motor status within the patient group were examined across MRC categories using linear models; the omnibus effect size is reported as partial eta squared (partial η^2^), with pairwise contrasts shown where applicable. Third, tumor-bearing (ipsilateral) and contralateral hemispheres were compared within patients using linear mixed-effects models to account for paired observations. These models included age, sex, and antiseizure medication (ASM) load as covariates, and the hemispheric effect is summarized by the standardized mean change. ASM load was calculated using the prescribed daily dose/defined daily dose (PDD/DDD) ratio [18].

Spatial analysis. Hotspot and CoG dispersion were evaluated independently of the above outcome models. Coordinates were transformed to common MNI space and mirrored to a common hemisphere for group-level comparison. Spatial dispersion was quantified from the spread of the covering ellipsoid (estimated as the average of the distances of each individual point to the ellipsoid centroid) and compared by permutation testing for (i) healthy controls versus patients and (ii) tumor-bearing versus contralateral hemispheres within patients.

Statistical reporting. Analyses were performed in MATLAB R2025b (MathWorks, Natick, MA, USA). All tests were two-sided, with an unadjusted significance threshold of *p* < 0.05. Normality was assessed using the Shapiro–Wilk test. Continuous variables are presented as mean ± standard deviation (SD) when normally distributed and as median (range) otherwise; categorical variables are presented as counts and percentages. Non-normally distributed outcomes were log-transformed when required for linear modeling. No missing values were imputed; each analysis used the available observations for the relevant outcome. Because 11 quantitative outcomes and several secondary contrasts were evaluated without formal multiplicity correction, secondary *p*-values are considered exploratory and are interpreted together with effect sizes and the consistency of findings across analyses. A non-significant result was not interpreted as evidence of equivalence.

## 3. Results

### 3.1. Demographic and Clinical Characteristics

Thirty-eight subjects were screened for eligibility. Twenty healthy volunteers and 18 patients completed the study per protocol. The mapping procedure was well tolerated; no participant required medical treatment, discontinued because of adverse effects, or reported adverse events.

FDI mapping was successfully performed in all subjects. DTI data were available for 13 healthy volunteers and 13 patients.

The healthy control group had a median age of 40 years (range 25–52) and comprised 10 females and 10 males; the patient group had a median age of 47 years (range 25–78) and comprised 9 females and 9 males. Demographic and clinical data are shown in Table 1 and Table 2.

### 3.2. Healthy Controls Versus Patients

After adjustment for age and sex, patients had lower grip strength, lower maximum FDI MEP amplitude, lower FDI map volume, and lower mean AD than healthy controls (Table 3). No statistically significant group differences were detected for FDI map area, NoF, mean FA, mean RD, fiber length, FAT75, or rMT. Age was associated with maximum FDI MEP amplitude and mean RD, while sex was associated with grip strength and FAT75. The adjusted rMT estimates were similar in magnitude (patients 40.82% versus controls 38.16% of maximum stimulator output; *p* = 0.5111). Given the number of outcomes examined, these between-group findings should be considered in terms of both effect size and the exploratory multiplicity of the analysis.

In the hemisphere-specific comparison with controls (available data from 14 patients for the hemisphere contralateral to the tumor), grip strength and maximum FDI MEP amplitude, FDI map volume, and mean AD differed in comparisons of healthy controls with both patient hemispheres, whereas reconstructed fiber length differed only in the comparison with the contralateral patient hemisphere (Table 4). These secondary comparisons were not adjusted for the full set of covariates and were not corrected for multiple testing; they are therefore descriptive/exploratory rather than confirmatory.

### 3.3. Association with Motor Grade (MRC)

**Figure 2:**
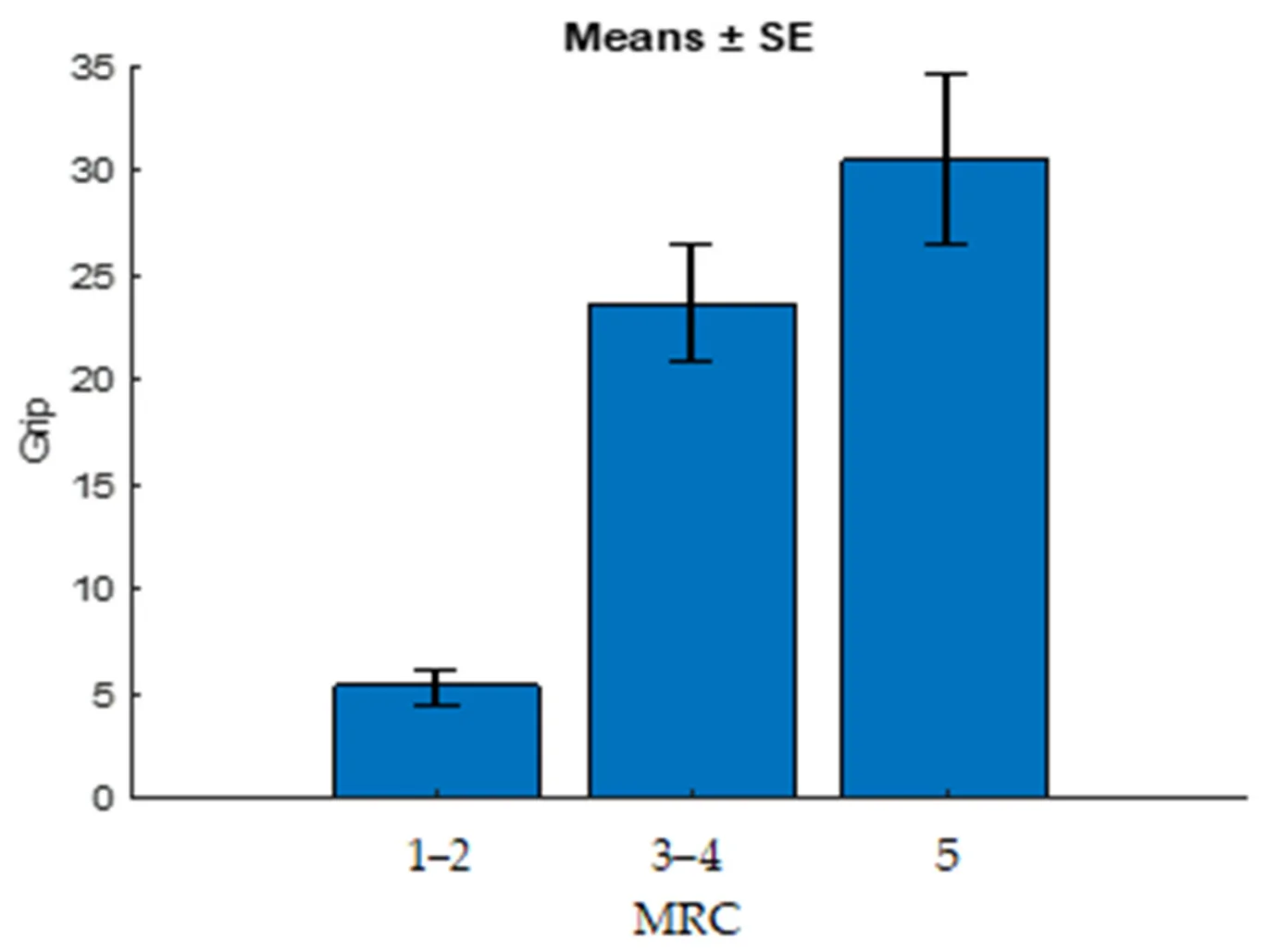
Grip strength according to MRC motor-grade category in patients. Bars show mean ± standard error. Grip strength differed significantly across MRC categories (overall *p* = 0.0002).

**Figure 3:**
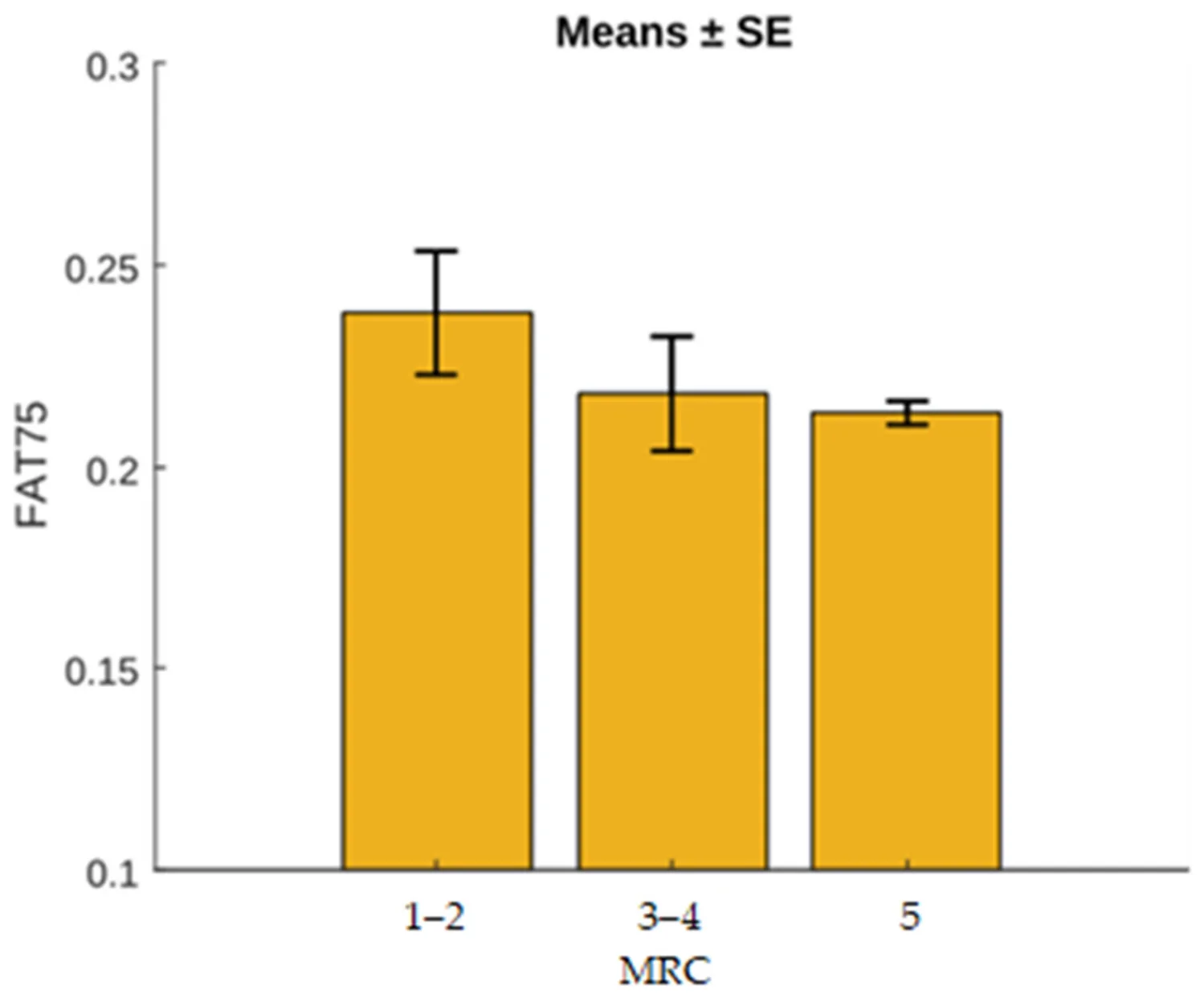
FAT75 according to MRC motor-grade category in patients. Bars show mean ± standard error. The omnibus comparison was not statistically significant (*p* = 0.0769); nominal pairwise *p*-values < 0.05 are exploratory.

MRC category was strongly associated with grip strength (overall *p* = 0.0002; partial η^2^ = 0.7512), providing expected convergent clinical evidence for the grip strength measurements (Figure 2). The overall test for FAT75 did not reach the conventional significance threshold (*p* = 0.0769), although pairwise contrasts were nominally significant for MRC one to two versus three to four (*p* = 0.0445) and MRC one to two versus five (*p* = 0.0364) (Figure 3). Because the omnibus test was non-significant and no multiplicity correction was applied, these FAT75 pairwise findings should be interpreted as hypothesis-generating. No other metric showed a statistically significant overall association with MRC grade (Table 5).

### 3.4. Ipsilateral Versus Contralateral Hemisphere in Patients

Within patients, grip strength was significantly lower for the tumor-affected side than for the contralateral side (*p* = 0.0001; standardized effect size = 1.21). No statistically significant hemisphere effect was detected for maximum FDI MEP amplitude, FDI map area or volume, rMT, or any nTMS-DTI metric (Table 6). Sex was associated with fiber length; age was associated with grip strength and fiber length; and ASM load was associated with mean FA, mean RD, and fiber length. These covariate associations should be interpreted cautiously because of the modest sample size and the number of modeled outcomes. As a sensitivity analysis addressing potential model instability and overfitting due to the small sample size, the models were refitted using hemisphere as the only predictor. The results were essentially unchanged: grip strength remained the only outcome showing a statistically significant hemispheric effect, and hemisphere-related *p*-values for the remaining outcomes changed only minimally.

### 3.5. Hotspot and Center-of-Gravity Spread

**Figure 4:**
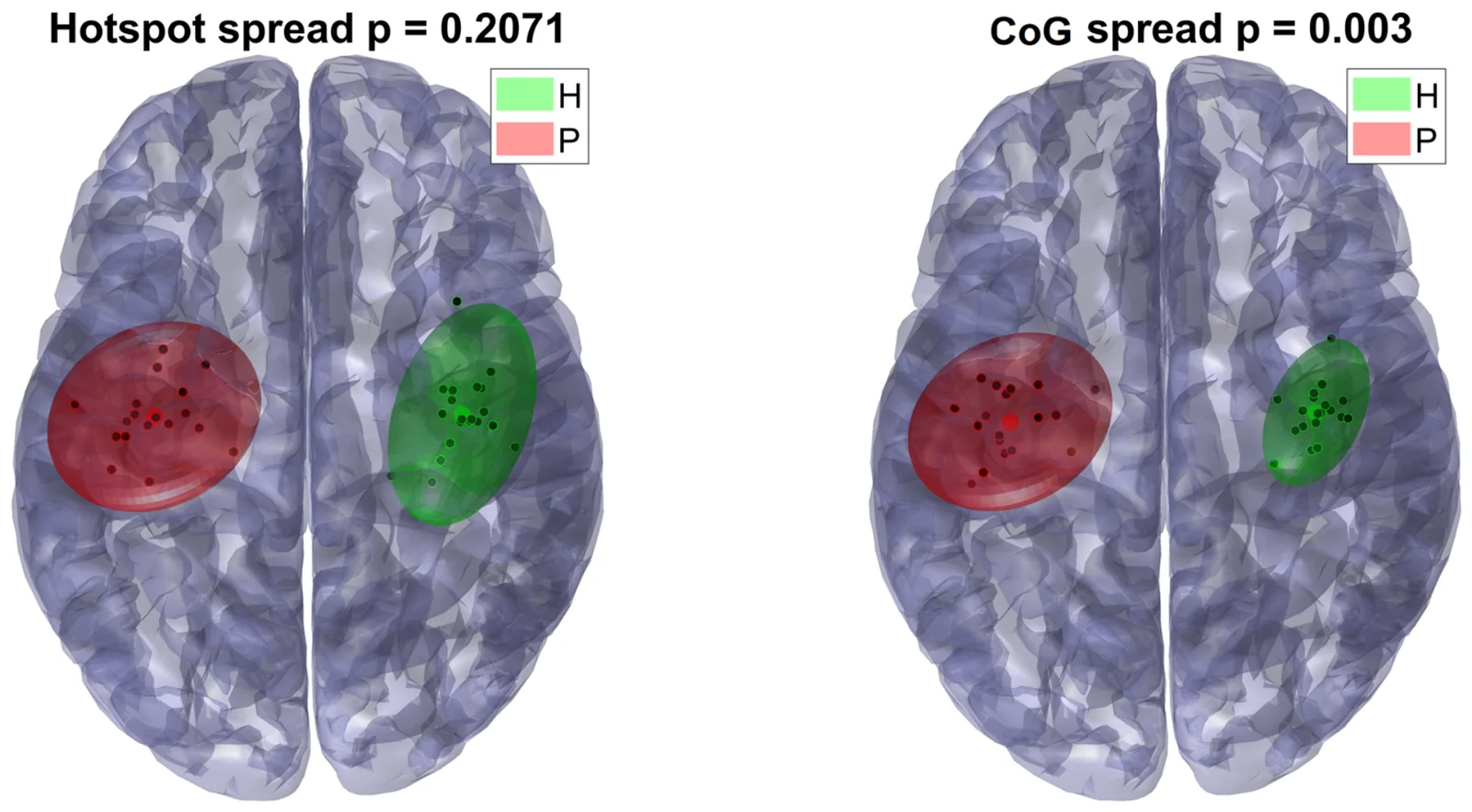
Spatial dispersion of the FDI hotspot and center of gravity (CoG) in healthy controls and patients after transformation to common MNI space and hemisphere normalization. CoG dispersion was greater in patients (permutation *p* = 0.0029), whereas hotspot dispersion did not differ significantly (*p* = 0.2071). Ellipsoids depict the spatial spread used in the permutation analysis. Individual hotspots and CoGs for each participant are depicted as black dots with green/red border for healthy/patient subjects. The group-level hotspot and CoG for healthy participants are represented by filled green dots, whereas those for patients are indicated by filled red dots.

**Figure 5:**
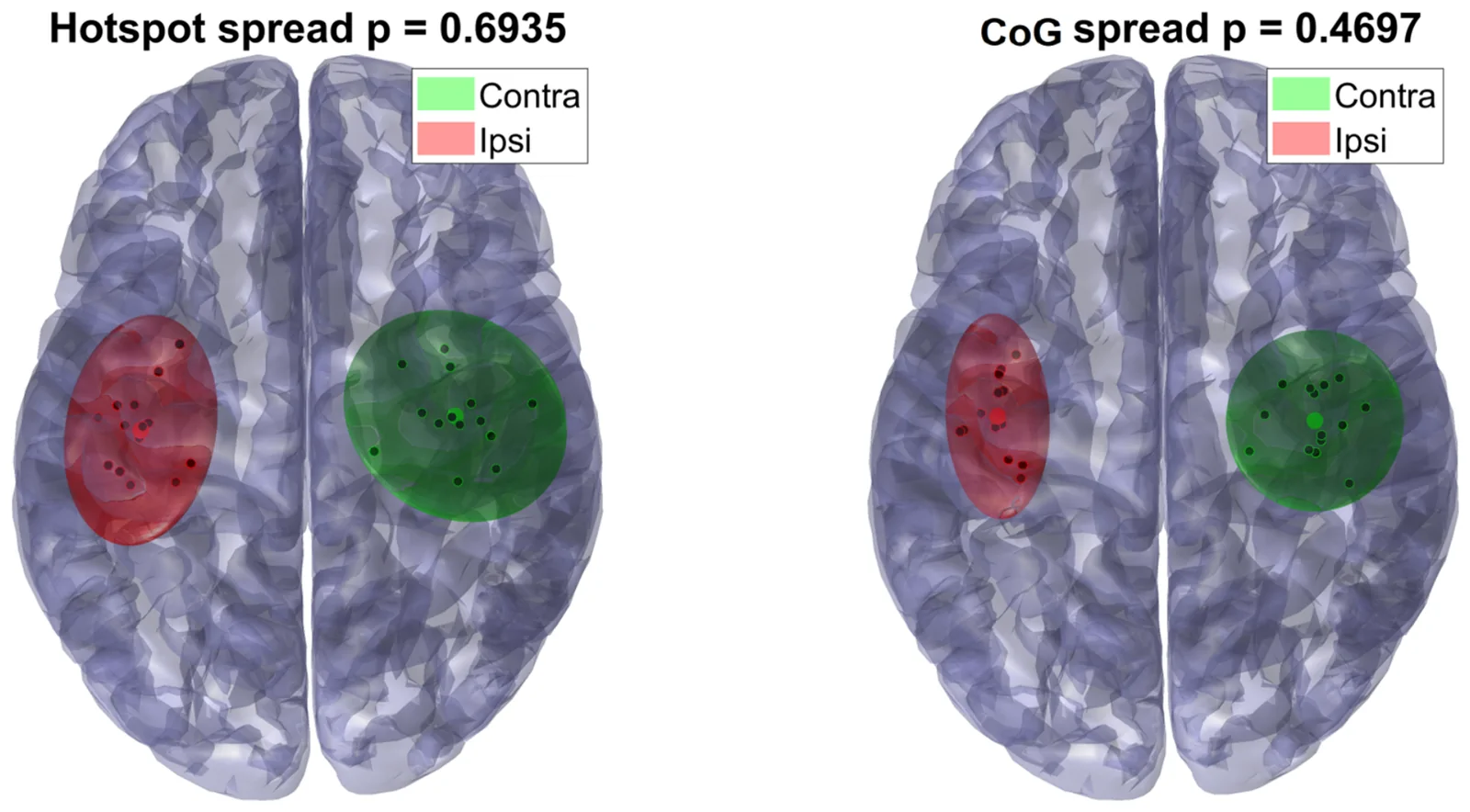
Spatial dispersion of the FDI hotspot and center of gravity (CoG) in the tumor-bearing (ipsilateral) and contralateral hemispheres after transformation to common MNI space. Neither hotspot nor CoG dispersion differed significantly between hemispheres. Ellipsoids depict the spatial spread used in the permutation analysis. Black dots with green/red border represent each contralateral/ipsilateral participant’s individual hotspot and CoG. Filled green dots show the overall (average) hotspot and CoG in the contralateral hemisphere, while filled red dots indicate those in the ipsilateral hemisphere.

The spatial dispersion of FDI CoGs was greater in patients than in healthy participants (10.872 vs. 5.705 mm, 95% CI of the difference (1.554, 7.986) mm, permutation *p* = 0.0029, effect size = 0.964), whereas hotspot dispersion did not differ significantly (11.035 vs. 8.383 mm, 95% CI of the difference (−1.491 6.562) mm, *p* = 0.2071, effect size = 0.425) (Figure 4). Within the patient cohort, neither hotspot nor CoG dispersion differed significantly between tumor-bearing and contralateral hemispheres (all *p* > 0.05, effect sizes = −0.1463 for hotspot and −0.3121 for CoG, 95% CIs of the difference (−5.835, 4.026) mm for hotspot and (−5.165, 2.499) mm for CoG) (Figure 5). Thus, the principal spatial finding was greater between-subject variability of the map’s amplitude-weighted center in patients, rather than a consistent within-patient displacement of the affected hemisphere.

## 4. Discussion

The findings can be considered in a hierarchy of confirmatory and potentially novel observations. First, the study confirms previous nTMS literature showing that tumors involving or adjacent to motor-eloquent structures are associated with impaired motor performance and altered suprathreshold corticospinal output. Compared with healthy controls, patients had lower grip strength, lower maximum FDI MEP amplitude, and lower FDI map volume. However, rMT was not significantly different, underscoring that individual excitability measures may be relatively insensitive to clinically meaningful motor system dysfunction. Second, and potentially more novel, abnormalities were not confined to the tumor-bearing hemisphere: the contralateral patient hemisphere also differed from healthy controls in grip strength, maximum FDI MEP amplitude, CST fiber length, and mean AD. Third, patients demonstrated greater between-subject CoG dispersion without a corresponding increase in hotspot dispersion. The latter two observations suggest that motor system alterations in patients with motor-eloquent tumors may extend beyond a simple focal ipsilesional abnormality and may involve bilateral/network-level changes and greater heterogeneity of cortical motor organization.

Given the multiplicity of outcomes, the principal patient–control findings were distinguished from the secondary and hypothesis-generating analyses, including hemisphere-specific, MRC-associated, and spatial comparisons.

### 4.1. Cortical Excitability, Motor Output, and Map Geometry

The reduction in motor performance and suprathreshold corticospinal output is consistent with previous nTMS studies of motor-eloquent brain tumors. In a cohort of 59 patients with diffuse glial tumors and 21 healthy controls, Naros et al. demonstrated impaired corticospinal integrity and emphasized that conventional MEP characteristics do not uniformly mirror the degree of motor dysfunction [19]. More recently, an analysis of 800 patients with motor-eloquent brain tumors showed that motor deficits were associated with higher rMT, reduced MEP amplitude, and prolonged MEP latency in the tumor hemisphere [20]. The present findings therefore reinforce an established observation: motor-eloquent tumors can impair motor function and suprathreshold corticospinal responsiveness. The contribution of the present dataset is not the demonstration of this impairment per se, but the simultaneous observation that some commonly used nTMS parameters, particularly rMT, may remain unrevealing at the group level.

The absence of a significant patient–control difference in rMT should not be considered contradictory to the clear reduction in grip strength and maximum FDI MEP amplitude. rMT represents the minimum stimulus intensity required to evoke a predefined motor response and predominantly reflects the excitability of the most readily recruited cortical and corticospinal neuronal elements. In contrast, suprathreshold MEP amplitude depends on recruitment and synchronization of a larger corticospinal neuronal population together with downstream spinal influences, while map volume integrates both spatial extent and response magnitude. Consequently, impaired motor system output may be expressed by lower suprathreshold MEP amplitude and map volume without a parallel change in rMT. This interpretation is supported by the substantial interindividual variability of rMT in brain tumor populations. Recent nTMS studies have shown that rMT is shaped by multiple clinical and anatomical factors, including lesion topography, motor deficit, tumor biology, edema, skull-to-cortex distance, and medication exposure [21,22]. In particular, precentral involvement and paresis have been associated with higher rMT, whereas high-grade glioma, motor-eloquent edema, and levetiracetam exposure have been associated with lower rMT in some cohorts [21]. Opposing influences within a heterogeneous patient group can therefore attenuate or obscure a group-level rMT difference.

ASM exposure adds a further interpretative constraint. Healthy controls were not taking centrally acting medication, whereas patients remained on stable ASMs. Pharmacological TMS studies demonstrate that ASMs can modify cortical excitability in a mechanism-dependent manner; sodium-channel-blocking agents such as carbamazepine and lamotrigine can increase motor threshold, whereas other agents may preferentially affect intracortical inhibitory or facilitatory circuits [23,24]. Thus, the similar mean rMT observed in patients and controls should not be interpreted as evidence of preserved intrinsic cortical excitability. Rather, rMT in an individual patient represents the net result of tumor location and biology, edema, network adaptation, seizure-related physiology, and pharmacological modulation. Because mapping intensity was normalized to each participant’s rMT (110% rMT), this individualized thresholding may nevertheless have helped compensate for interindividual differences and allowed reliable motor maps to be generated despite heterogeneous underlying physiology. Clinically, therefore, the important conclusion is that rMT can remain measurable and useful for mapping even when motor performance and suprathreshold corticospinal output are impaired—not that cortical excitability is normal.

### 4.2. Potentially Novel Finding: Increased CoG Dispersion

The greater CoG dispersion in patients, in the absence of a significant difference in hotspot dispersion, is also noteworthy. The two spatial measures capture different properties of the motor representation. In the present analysis, the hotspot was not the initial online site used for rMT determination; it was recalculated offline from the complete map and defined as the cortical location at which the averaged MEPs exhibited the highest peak-to-peak amplitude. It therefore represents a single maximum of averaged corticospinal output. In contrast, the CoG incorporates the position and response amplitude of the motor-positive sites across the map and may consequently be more sensitive to distributed changes in map organization. A motor representation may therefore become more spatially heterogeneous while retaining a relatively stable location of maximal averaged output. Prior nTMS studies have reported tumor-associated redistribution of motor-positive sites and changes in motor map topography [25,26]. Methodological differences in hotspot definition may contribute to discrepancies between studies: approaches based on the initial online hotspot or the site producing a maximal individual response are not directly equivalent to the post hoc TMSmap-derived hotspot based on averaged MEP responses used here. Accordingly, the absence of a significant hotspot dispersion difference in the present study should not be interpreted as evidence that hotspot topography is unaffected by motor-eloquent tumors. Rather, it may partly reflect the analytical definition used, whereas the CoG may capture broader redistribution of the motor representation. Nevertheless, the cross-sectional design cannot distinguish true neuroplastic reorganization from interindividual anatomical variability, lesion-related distortion, altered excitability, or differences in sampling. The CoG finding is therefore best interpreted as increased heterogeneity of motor map organization rather than direct proof of cortical plasticity.

### 4.3. Potentially Novel Finding: Bilateral Motor System Alterations and the Contralateral Hemisphere

A particularly striking and potentially novel observation was that patient–control differences were not restricted to the tumor-bearing hemisphere. When the two patient hemispheres were compared separately with healthy controls, the contralateral hemisphere was also associated with lower grip strength and lower maximum FDI MEP amplitude, together with differences in CST fiber length and mean AD. Although these comparisons were secondary and uncorrected for multiplicity, the pattern challenges the widespread assumption that the contralateral hemisphere in a patient with a unilateral motor-eloquent tumor necessarily constitutes a physiologically normal internal control. This issue is especially important because many nTMS studies characterize tumor-related excitability using interhemispheric ratios or the contralateral hemisphere as the reference.

Several non-mutually exclusive mechanisms may contribute to this bilateral phenotype. Gliomas can perturb distributed motor networks rather than only the cortex immediately adjacent to the lesion. Resting-state fMRI has demonstrated altered interhemispheric motor connectivity in patients with gliomas near motor areas [27], while structural network studies show that gliomas can alter connectivity beyond the immediate tumor environment [28]. nTMS studies likewise demonstrate tumor-associated redistribution and reorganization of motor representations [25,26]. Notably, the recent 800-patient nTMS study identified enlargement of the cortical motor area not only in the tumor hemisphere but, for oligodendrogliomas, also in the contralateral hemisphere [20]. These observations provide biological plausibility for bilateral motor system adaptation, although the mechanisms and functional significance remain incompletely defined.

Patient-level systemic factors may also contribute. Stable ASM exposure was present only in the patient group and could reduce or otherwise modify cortical responsiveness bilaterally. In addition, tumor-related functional limitation, seizure history, corticosteroid or other concomitant treatment, physical activity, and general disease burden may affect motor performance. The lower contralateral grip strength relative to healthy controls should therefore not be attributed automatically to structural disease of the contralateral corticospinal pathway.

The tractography findings require similarly cautious interpretation. Mean AD and reconstructed fiber length differed between healthy controls and both patient hemispheres, yet neither measure differed significantly between the two hemispheres within patients. This pattern is more consistent with a patient-level or bilateral effect than with a purely focal asymmetry caused by direct tumor involvement. However, AD is biologically non-specific in the setting of tumors, edema, compression, and infiltration, and tract length is a property of the reconstructed streamline set rather than a direct measure of axonal length. Tracking thresholds, ROI placement, crossing fibers, displacement, and premature streamline termination can all influence the estimated tract length. Consequently, these observations should not be interpreted as demonstrating contralateral axonal degeneration. They are better regarded as hypothesis-generating evidence that the motor system of patients with motor-eloquent tumors may differ from that of healthy individuals beyond the structurally affected hemisphere.

This finding has an important methodological implication. Studies relying exclusively on the contralateral hemisphere as a ‘normal’ comparator may underestimate patient-related abnormalities that are bilateral or systemic. The inclusion of an independent healthy control group, as in the present study, therefore provides complementary information that cannot be obtained from within-patient comparisons alone.

### 4.4. nTMS-Guided CST Tractography

At the whole-group level, most nTMS-DTI indices did not differ significantly between patients and controls, and no tractography metric showed a significant ipsilateral-versus-contralateral effect within patients. However, the hemisphere-specific comparison with healthy controls revealed differences in mean AD and fiber length for both patient hemispheres. These findings should be interpreted together: the lack of within-patient asymmetry does not imply normal tractography metrics, because both hemispheres may deviate from the healthy reference. At the same time, nTMS-seeded deterministic CST reconstruction remained technically feasible across the cohort, including in patients with weakness.

The reduction in mean AD and the shorter reconstructed fiber length relative to healthy controls deserve caution. AD can change with axonal injury, but its direction and biological meaning in brain tumors are non-specific and may be influenced by edema, cellularity, tract compression, infiltration, and acquisition characteristics. Fiber length is likewise reconstruction-dependent and can be altered by tracking thresholds, ROI placement, fiber displacement, crossing fiber configurations, and premature termination. Moreover, DTI data were available in only 13 controls and 13 patients, and multiple diffusion metrics and secondary comparisons were tested. These findings should therefore be considered exploratory rather than evidence of a specific bilateral microstructural mechanism. Similarly, the nominal FAT75 pairwise differences across MRC categories occurred despite a non-significant omnibus test and require independent confirmation.

ASM load was associated with several DTI outcomes in the within-patient models. A direct pharmacological effect on diffusion metrics cannot be inferred from these cross-sectional data; ASM load may also act as a marker of tumor-related seizure burden, lesion characteristics, or other clinical factors. Given the small number of patients relative to the number of covariates, these associations should be regarded as potential confounding signals rather than mechanistic findings.

### 4.5. Clinical Implications

From a clinical perspective, the results distinguish an established clinical observation from two potentially informative extensions. The confirmatory component is that motor-eloquent tumors are associated with impaired motor performance and reduced suprathreshold corticospinal output. The potentially novel observations are that patient–control abnormalities may extend to the contralateral hemisphere and that patients exhibit greater CoG dispersion, indicating increased heterogeneity of motor map organization. Reliable rMT estimation and motor mapping remained feasible despite these abnormalities. However, preserved or unrevealing rMT values should not be equated with normal cortical physiology, and the contralateral hemisphere should not automatically be regarded as a normal reference. Independent healthy controls may therefore add important information to studies of tumor-related motor system physiology and plasticity.

nTMS should therefore be viewed as complementary to structural imaging, tractography, neurological examination, and—when indicated—intraoperative mapping rather than as a stand-alone surrogate for functional integrity. The value of nTMS lies in providing individualized, spatially explicit functional information before surgery. Integration with CST tractography may provide complementary anatomical context for interpreting cortical motor representations; however, tractography parameters require standardized acquisition and tracking procedures and should be interpreted in the context of lesion biology [11,12,13,29]. These findings therefore support the role of nTMS primarily as a tool for physiological characterization and individualized functional mapping.

While our study was not specifically designed to identify the optimal parameters for nTMS application, our results endorse the utilization of standardized yet personalized neuronavigated methodologies as a foundation for future refinement, especially in patients exhibiting altered anatomical structures or tumor-induced cortical reorganization. This consideration is especially pertinent given recent evidence from Lucarelli et al. [30], which underscores the impact of technical TMS parameters on both physiological and functional outcomes of stimulation. In our research, the combination of nTMS-derived motor maps with DTI-based tractography furnished an additional structural perspective on the organization of the motor system, thereby enabling the investigation of the relationship between cortical motor representations and the underlying corticospinal pathways. Such an approach holds particular promise in clinical populations, where structural lesions and neuroplastic adaptations may modify the relationship between cortical representations and subcortical motor pathways. Future investigations should therefore focus on whether the integrated assessment of TMS-derived cortical maps and individual structural connectivity, in conjunction with systematic optimization of stimulation parameters, can enhance the accuracy, reproducibility, and clinical utility of TMS mapping and therapeutic administration.

### 4.6. Limitations

Several limitations materially affect interpretation. First, the sample was small (20 controls and 18 patients), and DTI was available for only 13 participants in each group; hemisphere-specific analyses included fewer patients as DTI acquisition was dependent on the clinical MRI protocol. The study was therefore underpowered for subtle effects, subgroup analyses, and robust multivariable modeling. Second, the patient cohort was clinically and pathologically heterogeneous. Tumor histology, grade, exact topography, volume, edema, infiltration, growth rate, seizure history, and treatment exposure can all influence cortical excitability and tractography, but these factors could not be modeled comprehensively.

Third, healthy participants underwent mapping of the dominant hemisphere, whereas patients underwent bilateral mapping. Hemisphere dominance and handedness may contribute to variability, and the patient–control comparison is therefore not a perfectly symmetric design. Fourth, medication exposure differed systematically between groups: healthy controls were free of centrally acting medication, whereas patients could receive stable ASMs. Because several ASMs can alter rMT and other TMS measures of cortical excitability [23,24], medication is an important potential confounder of the patient–control neurophysiological comparison. The present sample was too small and pharmacologically heterogeneous to model individual ASM mechanisms, doses, serum concentrations, or combinations adequately.

Fifth, multiple correlated outcomes and several pairwise comparisons were tested without a formal correction for multiplicity. Accordingly, the AD, fiber length, FAT75, and hemisphere-versus-control findings should be considered exploratory and require replication. Sixth, the cross-sectional design cannot establish neuroplastic reorganization or determine whether contralateral abnormalities predated the tumor, developed as compensatory network responses, or reflect systemic/pharmacological effects. Longitudinal mapping is required to demonstrate within-individual change. Seventh, hotspot definition is not uniform across the nTMS literature. The present topographical analysis used a post hoc TMSmap-derived hotspot based on averaged MEP amplitudes rather than the initial online hotspot used for rMT determination. Direct comparison of hotspot location or dispersion with studies using different hotspot definitions should therefore be made cautiously. To minimize the potential influence of sampling density on motor map parameters, motor maps were reconstructed using TMSmap software, which merges stimulation points separated by less than 2 mm before calculation of map area, volume, and CoG. Nevertheless, variations in spatial sampling and coverage cannot be entirely ruled out as potential sources of variability in these measures.

The DTI findings should be interpreted with caution due to the limited availability of DTI data and the methodological constraints inherent in deterministic tractography when applied to brain tumors. Furthermore, AD and reconstructed fiber length are metrics dependent on the specific model and algorithm employed; hence, they should not be regarded as direct indicators of axonal integrity or degeneration [31]. Consequently, observed bilateral differences in these parameters should be interpreted as variations in diffusion- and tractography-derived characteristics rather than conclusive evidence of bilateral corticospinal tract pathology.

Finally, there was no direct validation against intraoperative DES or postoperative functional outcomes in the present analysis, limiting conclusions about mapping accuracy or prognostic value.

## 5. Conclusions

This study confirms previous nTMS evidence showing that patients with motor-eloquent brain tumors exhibit impaired motor performance and altered suprathreshold corticospinal output, reflected here by lower grip strength, maximum FDI MEP amplitude, and FDI map volume relative to healthy controls. In contrast, rMT did not differ significantly, illustrating that a threshold excitability measure may remain unrevealing despite clinically and physiologically evident motor system dysfunction. Interpretation of rMT is further complicated by the opposing effects of lesion topography, tumor biology, edema, motor status, and ASM exposure. Beyond this confirmatory finding, two observations may represent more distinctive contributions of the present study: abnormalities in motor performance, suprathreshold MEP output, mean AD, and reconstructed fiber length were also detected when the contralateral patient hemisphere was compared with healthy controls, and patients demonstrated greater between-subject CoG dispersion. These findings raise the possibility that motor system alterations in brain tumor patients extend beyond the tumor-bearing hemisphere and reinforce the value of independent healthy controls rather than assuming that the contralateral hemisphere is physiologically normal. However, the presence of functional and tractography-derived differences in the contralateral hemisphere warrants cautious interpretation. Such abnormalities may reflect bilateral network reorganization or other clinical factors, including antiseizure medication exposure, rather than direct contralateral CST pathology. Accordingly, the contralateral hemisphere should not automatically be considered a physiologically normal reference for nTMS studies in patients with brain tumors. Larger longitudinal studies incorporating detailed medication exposure, tumor characteristics, standardized diffusion imaging, correction for multiple testing, intraoperative DES, and postoperative outcomes are required to determine the clinical significance and reproducibility of these potentially bilateral and spatially distributed changes.

## Figures and Tables

**Table 1:** Baseline demographic and clinical characteristics.

Characteristic	Patients	Healthy Controls	*p*-Value
No. of subjects, *n*	18	20	—
Female, *n* (%)	9 (50%)	10 (50%)	—
Male, *n* (%)	9 (50%)	10 (50%)	—
Age, median (range), years	47 (25–78)	40 (25–52)	0.1426 (z = 1.4660)
Handedness (EHI)			
Left	12 (60%)	15 (83%)	
Right	8 (40%)	3 (17%)	
Drug load, mean ± SD	1.7 ± 0.8	—	—
MRC grade 1, *n* (%)	1 (6%)	—	—
MRC grade 2, *n* (%)	6 (33%)	—	—
MRC grade 3, *n* (%)	1 (6%)	—	—
MRC grade 4, *n* (%)	6 (33%)	—	—
MRC grade 5, *n* (%)	4 (22%)	20 (100%)	—

MRC: Medical Research Council; SD: standard deviation; EHI: Edinburgh Handedness Inventory.

**Table 2:** Individual demographic and clinical characteristics of brain tumor patients.

Case	Sex	Age (Years)	MRC	Tumor Location	WHO Grade	ASM Load
1	F	47	4	R Temporal/Insular	Grade II	LEV: 2, LCM: 0.6
2	M	48	1	R Frontal/Parietal	Grade IV	LEV: 1.3
3	M	63	3	L Temporal	Grade IV	LEV: 1.3
4	F	67	2	L Frontal/Parietal	Grade III	BRV: 2, LCM: 1.3, PER: 0.5
5	F	54	5	L Temporal	Grade III	LEV: 2
6	M	58	2	L Frontal/Parietal	Grade IV	LEV: 1.3
7	M	26	5	R Frontal/Parietal	Grade II	LEV: 1.3
8	F	40	2	L Frontal/Temporal	Grade II	LEV: 0.3
9	F	27	4	L Frontal/Temporal	Grade II	LEV: 0.6
10	M	27	4	R Frontal/Temporal	Grade II	LCM: 1.3
11	M	50	4	L Temporal	-	LEV: 0.6, ESC: 1
12	F	39	5	L Frontal/Temporal	Grade III	LEV: 0.6
13	M	52	2	R Temporal	Grade II	LEV: 2
14	F	47	4	R Temporal/Parietal	Grade III	LEV: 2, LCM: 0.6
15	F	30	5	L Frontal/Temporal	Grade III	LEV: 1.3, LCM: 0.6
16	F	56	2	L Parietal	Grade III	LEV: 0.6
17	M	78	2	L Frontal/Parietal	Grade IV	LEV: 2
18	M	25	4	L Frontal	Grade II	LEV: 2

MRC, Medical Research Council; ASM: antiseizure medication; F: female, M: male; R: right; L: left; LEV: levetiracetam; LCM: lacosamide; BRV: brivaracetam; PER: perampanel; ESC: eslicarbazepine.

**Table 3:** Marginal means, model *p*-values, effect size, and 95% CI for the difference in the marginal means for healthy controls (H) versus patients (P), adjusted for sex and age.

Metric	Patients	Controls	*p* (Sex)	*p* (Age)	*p* (Condition)	Effect Size (Condition)	95% CI for Diff.
Grip (kg)	19.12	38.25	0.0009	0.0678	<0.0001	−1.8109	(−26.43, −11.81)
Max FDI	616.2	1834.5	0.2523	0.0342	0.0009	−1.2396	(−2382.09, −328.80)
Area (cm^2^)	4.05	5.67	0.3314	0.1490	0.1324	−0.5248	(−3.69, 0.92)
Volume (uV·cm^2^)	664.8	1667.4	0.3839	0.0739	0.0247	−0.7999	(−1473, −299)
NoF	46.83	64.69	0.5685	0.4315	0.4411	−0.3380	(−65.08, 29.35)
FA mean	0.5397	0.5742	0.5000	0.1111	0.0600	−0.8543	(−0.07, 0.01)
AD mean	0.0013	0.0014	0.7226	0.2246	0.0045	−1.3632	(−0.00012, −0.00002)
RD mean	0.0005	0.0005	0.5064	0.0230	0.2454	0.5141	(−0.00002, 0.00006)
Fiber length	150.36	161.21	0.3576	0.8060	0.1013	−0.7368	(−24.00, 2.30)
FAT75	0.2228	0.2388	0.0275	0.3175	0.2290	−0.5330	(−0.04, 0.01)
rMT	40.82	38.16	0.7791	0.0847	0.5111	0.2260	(−5.49, 10.82)

AD: axial diffusivity; FA: fractional anisotropy; FAT75: 75% of the subject-specific FA threshold; FDI: first dorsal interosseous; NoF: number of fibers; RD: radial diffusivity; rMT: resting motor threshold. Values are adjusted marginal means from patient–control linear models including age and sex. Effect size is the adjusted standardized mean difference; the sign follows model coding. No multiplicity correction was applied.

**Table 4:** Condition *p*-values comparing controls with the ipsilateral and contralateral patient hemispheres. The first column is the same as *p* (Condition) in Table 3.

Metric	*p* (H vs. P Ipsilateral)	*p* (H vs. P Contralateral)
Grip	<0.0001	0.0043
Max MEP	0.0009	0.0293
Area	0.1324	0.0593
Volume	0.0247	0.0472
No. of fibers	0.4411	0.7906
FA mean	0.0600	0.4400
AD mean	0.0045	0.0052
RD mean	0.2454	0.7583
Fiber length	0.1013	0.0362
FAT75	0.2290	0.6418

H: healthy controls; P: patients; other abbreviations as in Table 2. Values are condition *p*-values for controls versus the ipsilateral or contralateral patient hemisphere. These secondary comparisons were not corrected for multiplicity; nonsignificant results do not establish equivalence.

**Table 5:** Patient metrics by MRC grade (overall and pairwise *p*-values; partial η^2^).

Metric	Overall	1–2 vs. 3–4	1–2 vs. 5	3–4 vs. 5	η^2^
Grip	0.0002	0.0006	0.0001	0.0270	0.7512
Max MEP	0.9601	0.9006	0.7836	0.8355	0.0068
Area	0.5220	0.8746	0.4231	0.2680	0.1027
Volume	0.7021	0.8096	0.6234	0.4102	0.0572
No. of fibers	0.2626	0.1644	0.7237	0.2266	0.3175
FA mean	0.6975	0.4139	0.6092	0.7626	0.0978
AD mean	0.3911	0.4391	0.1899	0.3827	0.2353
RD mean	0.6731	0.5997	0.8697	0.4188	0.1070
Fiber length	0.9005	0.6656	0.8226	0.8308	0.0295
FAT75	0.0769	0.0445	0.0364	0.6242	0.5196
rMT	0.2504	0.8339	0.2284	0.1064	0.2061

Abbreviations as in Table 2. η^2^ is partial eta squared for the omnibus MRC-grade effect. Pairwise *p*-values are unadjusted. Because the FAT75 omnibus test was nonsignificant (*p* = 0.0769), nominally significant FAT75 pairwise contrasts are exploratory.

**Table 6:** Patient ipsilateral versus contralateral hemisphere: marginal means, model *p*-values, hemisphere effect size and 95% CI for the difference in the marginal means.

Metric	Ipsi	Contra	*p* (Sex)	*p* (Age)	*p* (ASM Load)	*p* (Ipsi/Contra)	Effect Size	95% CI for Diff.
Grip (kg)	10.98	21.62	0.0880	0.0096	0.0766	0.0001	1.2094	(−15.65, −5.84)
Max FDI	1279.97	928.90	0.1991	0.3782	0.9612	0.3451	0.2482	(−646.91, 506.63)
Area (cm^2^)	4.41	3.88	0.6150	0.8835	0.0980	0.7958	−0.0674	(−2.89, 2.26)
Volume (uV·cm^2^)	1247.24	856.01	0.4120	0.8320	0.2793	0.8744	0.0412	(−1325.60, 1667.52)
No. of fibers	53.13	48.30	0.9917	0.8660	0.4416	0.6388	−0.1437	(−41.98, 64.98)
FA mean	0.5509	0.5510	0.1522	0.7898	0.0025	0.4405	0.2377	(−0.03, 0.01)
AD mean	0.0013	0.0013	0.2446	0.0696	0.6015	0.7151	−0.1116	(−0.00003, 0.00005)
RD mean	0.0005	0.0005	0.4256	0.7152	0.0007	0.1483	−0.4572	(−0.00001, 0.00003)
Fiber length	147.30	152.15	0.0064	0.0031	0.0001	0.6359	−0.1450	(−7.71, 11.98)
FAT75	0.2328	0.2259	0.2596	0.4343	0.1059	0.9444	−0.0213	(−0.03, 0.03)
rMT	35.53	31.78	0.9901	0.8294	0.2108	0.9843	0.0051	(−7.76, 7.61)

AD: axial diffusivity; ASM: antiseizure medication; Contra: contralateral; FA: fractional anisotropy; FAT75: 75% of the subject-specific FA threshold; FDI: first dorsal interosseous; RD: radial diffusivity; rMT: resting motor threshold; Ipsi: ipsilateral. Linear mixed-effects models account for paired hemispheric observations and include sex, age, and ASM load as covariates. Effect size is the standardized mean change for the hemispheric contrast. Nonsignificant hemisphere effects do not establish equivalence.

## Data Availability

The raw data supporting the conclusions of this article will be made available by the authors on request.

## Abbreviations

The following abbreviations are used in this manuscript:

AD	Axial Diffusivity
ASM	Antiseizure medication
BRV	Brivaracetam
CoG	Center-of-gravity
CST	Corticospinal Tract
DDD	Defined Daily Dose
DES	Direct Electrical Stimulation
DTI	Diffusion Tensor Imaging
EHI	Edinburgh Handedness Inventory
EMG	Electromyography
ESC	Eslicarbazepine
FA	Fractional Anisotropy
FAT75	75% of the subject-specific FA threshold
FDI	First Dorsal Interosseous
LEV	Levetiracetam
LCM	Lacosamide
MRC	Medical Research Council
MCR	Muscle Cortical Representation
MEP	Motor Evoked Potential
MRI	Magnetic Resonance Imaging
NoF	Number of Fibers
nTMS	Navigated Transcranial Magnetic Stimulation
PDD	Prescribed Daily Dose
PER	Perampanel
RD	Radial Diffusivity
ROI	Region-of-interest
rMT	Resting Motor Threshold
SD	Standard Deviation
TMS	Transcranial Magnetic Stimulation
